# 3D-Printed Drug Delivery Systems: The Effects of Drug Incorporation Methods on Their Release and Antibacterial Efficiency

**DOI:** 10.3390/ma13153364

**Published:** 2020-07-29

**Authors:** Bahaa Shaqour, Inés Reigada, Żaneta Górecka, Emilia Choińska, Bart Verleije, Koen Beyers, Wojciech Święszkowski, Adyary Fallarero, Paul Cos

**Affiliations:** 1 Voxdale bv, Bijkhoevelaan 32C, 2110 Wijnegem, Belgium; bart@voxdale.be (B.V.); koen@voxdale.be (K.B.); 2 Laboratory for Microbiology, Parasitology and Hygiene (LMPH), Faculty of Pharmaceutical, Biomedical and Veterinary Sciences, University of Antwerp, Universiteitsplein 1 S.7, 2610 Antwerp, Belgium; paul.cos@uantwerpen.be; 3 Drug Research Program, Division of Pharmaceutical Biosciences, Faculty of Pharmacy, University of Helsinki, 00790 Helsinki, Finland; ines.reigada@helsinki.fi (I.R.); adyary.fallarero@helsinki.fi (A.F.); 4 Faculty of Materials Sciences and Engineering, Warsaw University of Technology, Woloska 141, 02-507 Warsaw, Poland; gorecka.zaneta@gmail.com (Ż.G.); choinska.emilia@gmail.com (E.C.); wojciech.swieszkowski@pw.edu.pl (W.Ś.)

**Keywords:** fused filament fabrication, 3D printing, drug loading, drug release

## Abstract

Additive manufacturing technologies have been widely used in the medical field. More specifically, fused filament fabrication (FFF) 3D-printing technology has been thoroughly investigated to produce drug delivery systems. Recently, few researchers have explored the possibility of directly 3D printing such systems without the need for producing a filament which is usually the feedstock material for the printer. This was possible via direct feeding of a mixture consisting of the carrier polymer and the required drug. However, as this direct feeding approach shows limited homogenizing abilities, it is vital to investigate the effect of the pre-mixing step on the quality of the 3D printed products. Our study investigates the two commonly used mixing approaches—solvent casting and powder mixing. For this purpose, polycaprolactone (PCL) was used as the main polymer under investigation and gentamicin sulfate (GS) was selected as a reference. The produced systems’ efficacy was investigated for bacterial and biofilm prevention. Our data show that the solvent casting approach offers improved drug distribution within the polymeric matrix, as was observed from micro-computed topography and scanning electron microscopy visualization. Moreover, this approach shows a higher drug release rate and thus improved antibacterial efficacy. However, there were no differences among the tested approaches in terms of thermal and mechanical properties.

## 1. Introduction

Additive manufacturing (AM), also known as 3D printing, has been extensively explored in the past decade. This technology has helped to pave the way for personalized and customized production. Fused deposition modeling (FDM™), also known as fused filament fabrication (FFF), is one of the 3D printing technologies that was widely used after the expiration of the Stratasys patent in 2009 [1]. As this technology is cheaper and more flexible than other 3D printing technologies, many studies explored its potential in fields such as personalized medicine [2]. The focus was on producing patient specific drug delivery systems, such as tablets for oral drug delivery or implants for localized drug delivery.

The typical approach for 3D printing a drug delivery system includes: (1) selection of a carrier polymer and a required active pharmaceutical ingredient (API); (2) preparing a filament using the hot melt extrusion (HME) process; (3) designing the geometry of the drug loaded device; and 4) 3D printing [3].

The step that involves the filament preparation can be omitted if a 3D printer was designed to be compatible with the polymer and the drug in their original form as a raw material. Interestingly, only a few studies have used such systems [4,5,6], while the vast majority of studies have used the previously mentioned approach. There are many reasons for the limited interest in systems that do not include the filament preparation step in the process. Among them is the well-established knowledge of HME technologies. Another reason is the complex optimization process and parameter setting in these 3D printers. This complexity is due to the extrusion system used in these printers, which can either be a screw-based or pneumatic extrusion system or both. Moreover, with this configuration a challenge arises, which is to achieve a homogenous mixture between the carrier polymer and the API, especially when the latter is much lower in volume or there is a large difference in size, such as pellets, chips, or powders. As there are countless approaches for pre-mixing the polymer and the drug, it was noticed that a standardized method for the loading of an API into a carrier polymer for 3D printing purposes is not available [7].

In this study, gentamicin sulfate (GS) was selected as a model drug. It is an aminoglycoside antibiotic used to treat several types of bone and soft tissue infections [8]. It has a high solubility in water [9]; however, its effectiveness is concentration-dependent, especially for the treatment of biofilms. These kinds of infections are often hospital-acquired and associated with medical device use, being up to 80% of these infections caused by bacterial biofilms [10]. Biofilms are defined as a community of cells encased within a self-produced matrix that adheres to biological or non-biological surfaces [11,12]. By switching into the biofilm state, bacteria can withstand antibiotic chemotherapy, increasing the minimum inhibitory concentration (MIC) up to 1000 times [13], which is increasingly regarded as the most important nonspecific mechanism of antimicrobial resistance [14,15]. This is especially true for orthopedic implants, where biofilm-associated infections cause up to 25% of failures [16].

The implantation of a foreign material represents *per se* a major risk factor for the development and chronic progression of osteomyelitis. Presently, the commonly used method of prevention is systemic administration of oral antibiotics, but this can result in adverse effects and the generation of bacterial resistance. Drug delivery systems have many advantages, among them is the delivery of API at the targeted physical site for a prolonged period and at the required therapeutic level [17]. Additionally, 3D printing can be used to produce drug eluting medical devices that may provide the proper concentration of API.

Moreover, the use of GS in this approach has proven efficacy because the system based on GS loaded into Poly(methyl methacrylate) (PMMA) beads is widely used for infection eradication in orthopedic applications. As carrier polymer to be investigated, polycaprolactone (PCL) was selected. This has been extensively explored for applications such as tissue engineering and drug-delivery systems due to its biocompatibility and biodegradability [18,19]. Loading PCL with antibiotics such as GS has shown great potential during recent years. Such drug delivery systems have been extensively explored among the literature [20,21]. It was shown that the produced systems can prevent bacterial infections and biofilm formation both in vitro and in vivo.

This study compares two mixing approaches widely used in the literature. The first approach is powder mixing. In this approach the carrier polymer and the drug are simply mixed physically; however, it is very important that they have similar forms in terms of geometry to assure homogenous mixing before 3D printing. Saviano et al. [22] discussed the effect of polymer size on the homogeneity of the drug distribution in drug loaded devices and also the extrudability of the mixture. They tested a range of polymer sizes from powders with less than 250 µm particle size up to pellets with diameters of 4 to 5 mm. They concluded that the moderate size particles, ranging from 250 to 600 µm, allow for homogeneous drug dispersion in the polymer matrix and good processability. On the other hand, a similar effect can be obtained by the second mixing method, i.e., the solvent casting technique. In this approach, an organic solvent is used to dissolve the polymer and then the drug is suspended in the system [23]. Subsequently, the solution/suspension is cast, and the solvent can evaporate. However, it includes the use of relatively large volumes of solvents, which can be toxic, and make the process not environmentally friendly.

The aim of this study was to shed light on the preparation methods of drug loaded printable materials. This very important issue has not been thoroughly discussed in the literature. As the production of drug loaded devices using 3D printing technologies has been intensively investigated over recent years, the methods of material preparation or drug incorporation have varied between the different studies. Additionally, there are no standardized methods of drug incorporation into a polymeric carrier prior to 3D printing, which makes it difficult to compare between published results. Moreover, with the new trend of single step 3D printing, it is very important to investigate the most effective drug loading approaches for this process. Thus, evaluation of material preparation techniques and the effects of different incorporation methods on drug distribution, release, and antibacterial efficiency is key for producing standard drug loading and incorporation techniques.

## 2. Materials and Methods

PCL pellets (Mn 80,000, cat no.: 440744-500G) were obtained from Sigma Aldrich (Gillingham, U.K.), GS, USP grade was obtained from Fluorochem Ltd. (Glossop, U.K.). Solvents used such as dichloromethane (DCM), chloroform, ethanol, and iso-propanol, and components to prepare the phthaldialdehyde reagent were obtained from Chempur (Piekary Śląskie, Poland). Tryptic Soy Broth (TSB) and tryptic soy agar (TSA) were obtained from Neogen^®^ (Lansing, MI, USA).

### 2.1. Preparation of Mixtures

PCL was loaded with GS at two concentrations 2.5% and 7.5% (*w/w*). In the solvent casting approach, the mixture was prepared by suspending the proper amount of drug (based on the required drug loading) in 100 mL of DCM using a magnetic stirrer (VELP Scientifica Srl, Usmate, Italy) for 10 min. Subsequently, 10 g of PCL was added and stirred overnight to form a 10% (*w/v*) solution. The polymer solution with suspended drug was then poured on a petri dish (ϕ 200 mm) to evaporate the solvent under the fume hood for two days. Finally, the films were vacuum dried for three days at 25 °C and 50 mbar. Then, 10 mm × 10 mm square pieces of drug loaded PCL were cut for printing.

**Figure 1:**
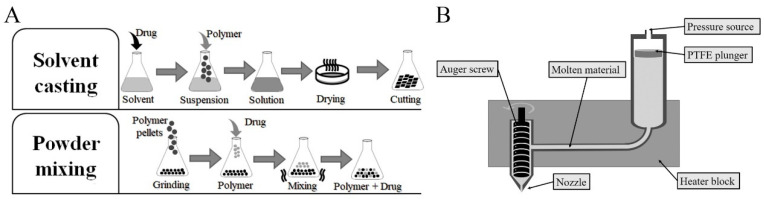
(**A**) Steps performed for preparing the drug-polymer mixture via solvent casting and powder mixing approaches and (**B**) The 3D printer extruder used for printing the samples.

In the powder mixing approach, the mixture was prepared by grinding PCL pellets using a grinder (Retsch ZM200, Katowice, Poland). First, pellets were frozen using liquid nitrogen, then grinded at 60,000 rpm and sieved (aperture 0.75 mm). The grinder was kept chilled by pouring small portions of liquid nitrogen during the grinding process. Subsequently, the polymer was added to 50 mL tubes. Then, the proper amount of drug was added, and the mixture was vortexed well for 5 min. Finally, the mixture was fed to a Bioscaffolder (SYSENG, Salzgitter, Germany). Figure 1A illustrates the two mixing approaches.

### 2.2. Preparation of Samples

**Figure 2:**
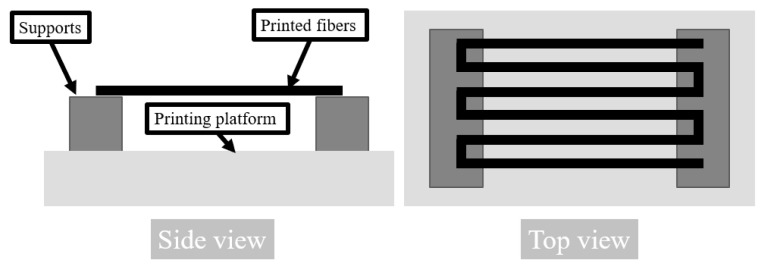
Setup for 3D printing of fibers.

The models of samples for printing were designed in SolidWorks (v.2017) software. Samples were 3D printed using a Bioscaffolder (SYSENG, Salzgitter, Germany) (printing head scheme presented in Figure 1B) and a dedicated Bioscaffolder-software (PrimCam v.3.0) for the slicing process. A nozzle with 1 mm diameter was used. The temperature of printing was set to 100 °C. The X and Y axes feed rate was set to 30 mm/min, the screw extruder rotation was set at 38 rpm and a pneumatic pressure of 5 bar was applied. Three types of samples were prepared. For µCT scanning and tensile testing, single fibers were extruded through the nozzle by printing infill of rectangular single layer of cuboid with 2 mm distance between each line. A special platform (Figure 2) for the printing bed was prepared, which enabled printing of around 1 mm of the start and the end of the line on the platform, while the rest was hung in the air [24]. For release tests, disk-shaped samples with a diameter and height of 10 mm and 2 mm respectively, were designed and printed directly on a printing platform. The infill density was set to 100%. The layer thickness was 0.8 mm. Samples for the compression test were cut out from similarly printed bulk material with a biopsy puncher to achieve a repeatable cylinder with a 4 mm diameter. The height of these samples was also 4 mm.

### 2.3. Scanning Electron Microscopy (SEM) Analysis

The images of drug and polymer particles were acquired using a scanning electron microscope (SEM) (Phenom Pro X, Phenom-World, Eindhoven, The Netherlands) equipped with a holder dedicated for testing of non-conductive samples. The 10 kV acceleration voltage was applied. Prior imagining samples were sputtered with a 7 nm conductive gold layer.

### 2.4. Micro-Computed Tomography (µCT)

µCT measurements were performed on the 2 mm-long fibers using SKYSCAN 1172 (Bruker, Kontich, Belgium). The scan was performed at 40 kV and 250 mA over 180 degrees with a rotation step of 0.5° and exposure time of 600 ms. The image pixel size was 1.46 µm. The obtained planar images were reconstructed and analyzed with the instrument software—NRecon and CTAn (v1.10.1.0, Bruker, Kontich, Belgium). Quantitative measurements and 3D models of GS were performed by segmentation of drug particles in 8-bit greyscale images. The particles of GS with pores inside were treated as a whole body for analysis of dimensions of particles. The dimensions of particles were calculated as a diameter of the maximal sphere fitted within single particles. The quantitative analysis was performed on six volumes of interests for each type of fibers.

### 2.5. Thermal Analysis

Thermogravimetric analysis (TGA) was conducted on a Q5000 Analyzer (TA Instruments, New Castle, DE, USA). Samples of around 10 mg were placed on a platinum pan and were then heated to 530 °C, at a rate of 10 °C/min under a nitrogen flow of 25 mL/min.

Differential Scanning Calorimetry (DSC) was conducted on a Q2000 apparatus (TA Instruments, New Castle, DE, USA). Samples of around 5 mg were sealed in an aluminum pan and heated in the range of −80 °C up to 80 °C, at a rate of 10 °C/min under a nitrogen flow of 50 mL/min. For GS samples, the range of temperature was from 20 °C to 200 °C, with a similar machine configuration. Three samples from the printed fibers were used for this analysis. Only one heating cycle was performed. The percentage of crystallinity of PCL was calculated based on the following formula:

$$Crystallinity=\frac{H_{s}}{H_{fc}*R}\cdot 100\%$$

where $H_{s}$ is the melting enthalpy of a sample, R is the PCL content in a sample and $H_{fc}$ is the melting enthalpy of 100% crystalline PCL which is 142 J/g [25].

### 2.6. Mechanical Testing

Tensile and compression tests were performed on MicroTester 5943 (Instron, Norwood, MA, USA). For tensile testing, the 20 mm-long fibers were used. The tensile rate was set to 0.1 mm/min and the distance between grips was 10 mm; according to guidelines about elastic modulus measurement in ISO527-1 the chosen tensile rate should provide 1% of strain per min. For compression testing, samples with 4 mm height and 4 mm diameter were tested at a rate of 0.5 mm/min. Five samples were tested for each analysis per group. The elastic modulus (EM) was calculated from the stress-strain curve generated by the machine’s software (BlueHill 3, v3.72, Instron, Norwood, MA, USA). A MATLAB code was written to calculate the slope of the best fit line between the 2.5% and 7.5% strain.

### 2.7. Drug Loading and Release Test

The drug loading test was performed for the first and last disks printed in the batch for the drug release experiment (20 samples in batch). The samples were dissolved in chloroform overnight. The solution was thoroughly mixed, and 1 mL aliquots were withdrawn and mixed with 5 mL of phosphate buffer solution (PBS), then stirred for an hour to assure the dissolution of GS in the PBS. The mixture was then left for 30 min without stirring until a clear phase separation was achieved. Finally, an aliquot from the PBS phase was removed and was used for measurements of the concentration of GS.

For the drug release test, three samples per group were sterilized by dipping in ethanol (96%), then the samples were dried in the laminar air flow cabinet (BioWizard Silver SL-200 Blue Series Class II, KOJAIR®, Mänttä-Vilppula, Finland) for a couple of hours and placed in 4.5 mL of PBS at 37.5 °C. At each time point the buffer solution was replaced with a fresh one. The collected PBS, with the extracted (released) GS, was used in measurements of concentration of GS.

In both experiments (drug loading and drug release), the GS assay was performed by mixing collected PBS aliquots with phthaldialdehyde reagent and iso-propanol in a 1:1:1 (*v/v*) ratio [26,27,28]. This mixture was left for 45 min to react. A calibration curve was prepared and was used to measure the content of the GS in the aliquots using a UV-VIS spectrometer Nicolet Evolution 60 (Thermo Fisher Scientific, Madison, WI, USA) at a 332 nm wavelength. The range used for the calibration curve was between 294.8 up to 2.3 ug/mL with R^2^ of 0.9984. All measure aliquots were in the selected range of detection.

### 2.8. Minimum Inhibitory Concentration (MIC)

The minimum inhibitory concentration—the lowest concentration of the eluate that completely inhibited visible growth of methicillin-sensitive *Staphylococcus (S.) aureus* (ATCC 25923) after 24 h of incubation at 37 °C—was determined in 96-well microplates. The serial 1:1 dilutions of the eluates were made in TSB at a volume of 90 μL/well in 96-well microtiter plates. Each well was then inoculated with 10 μL of the bacterial inoculum. To prepare the inoculum, *S. aureus* (ATCC 25923) was cultured in 30 g/L TSB under aerobic conditions at 37 °C, 220 rpm for 4 h to reach the exponential phase (around 10^8^ CFU/mL). This was estimated by spectrophotometric turbidity measurements at 595 nm using a Thermo Scientific Multiskan Sky microplate spectrophotometer (Thermo Fisher Scientific, Waltham, MA, USA) and by CFU counts on TSA plates. The concentration was then adjusted to 10^7^ CFU/mL. The effect was examined after incubation at 37 °C at 200 rpm for 24 h in a humidified incubator.

### 2.9. Minimum Biofilm Inhibitory Concentration (MBIC)

The anti-biofilm effects of the eluates were assessed prior to biofilm formation. This was carried out as described earlier by Fallarero et al. [29] with some modifications. One modification was that given the large number of samples, 348-well microplates were used instead of 96-well microplates [30]. The bacterial inoculum was prepared as described in Section 2.8, and adjusted to a concentration of 10^6^ CFU/mL. To form biofilms, these exponentially grown cultures (10^6^ CFU/mL) were added into flat-bottomed 348-well microplates (Nunclon Δ surface, Nunc, Roskilde, Denmark) simultaneously with the eluates at a 1:1 ratio, in a final volume of 40 µL. As a negative control, a PBS-TSB solution was used at a ratio 1:1, in a final volume of 40 µL. The biofilm inhibitory effect was examined after incubation at 37 °C, 200 rpm for 18 h.

The effect on the biofilm viability was measured by following the protocol by Skogman et al. [31], using resazurin redox staining. Briefly, the planktonic suspension was removed and biofilms were washed twice with 50 µL of PBS, then they were stained with 20 µM resazurin for 20 min at 200 rpm at room temperature, and the fluorescence was measured at ʎ_excitation_ = 560 nm and ʎ_emission_ = 590 nm (Varioskan LUX Multimode Microplate Reader, Thermo Scientific, Waltham, MA, USA).

### 2.10. Adherence of Bacteria to the Surface

The adherence of bacteria was evaluated by the static biofilm method with some modifications according to the protocol by Hiltunen et al. [32]. A sterile Whatman filter paper (70 mm diameter, qualitative grade 2, GE Healthcare, Little Chalfont, U.K.) was placed on TSA plate (90 × 15 mm). For biofilm formation, the pre-culture of methicillin-sensitive *S. aureus* (ATCC 25923)—1 mL of colonies suspended into 100 mL of TSB—were grown under aerobic conditions at 37 °C and 220 rpm for 20 h, and then diluted 1:10 in TSB to obtain approximately 10^8^ CFU/mL of bacteria. The filter paper was inoculated with 1.5 mL of the bacterial dilution. This was followed by the addition of the printed materials similar to the ones used for the drug release test, three samples corresponding to each mixing approach and each dosage of 2.5 and 7.5%. As a negative control, PCL printed samples were used. This assembly was incubated under humidified aerobic conditions at 37 °C for 24 h. After incubation, samples were washed with TBS to remove any remaining planktonic cells and then they were transferred into falcon tubes containing 1 mL of 0.5% (*w/v*) Tween20:TSB solution. Subsequently, the tubes were sonicated in a water sonicator (Ultrasonic Cleaner 3800, Branson Ultrasonics, Danbury, CT, USA) at 25 °C, for 5 min at 35 kHz. The tubes were mixed vigorously for 20 s prior to and after the sonication step. Serial dilutions were performed from the resulting bacterial suspensions and plated on TSA (Neogen^®^, Lansing, MI, USA) plates.

### 2.11. Statistical Analyses

Quantitative data were expressed as an average ± standard deviation, number of samples are stated in each experiment. The statistical analyses were performed using the SPSS v.26 software and a one-way analysis of variance (ANOVA). TukeyHSD methods was used to measure statistical significance between groups, *p* < 0.05 was considered to be statistically significant.

## 3. Results and Discussion

**Figure 3:**
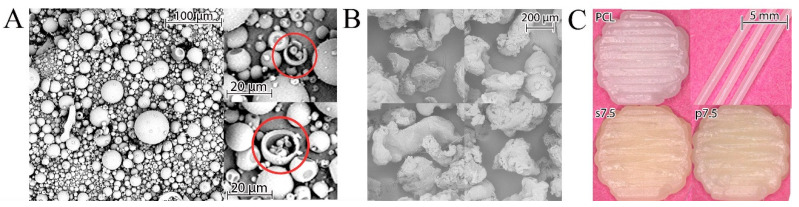
(**A**) SEM images of GS, red circles indicate some fractured spheres showing their hollow structure, (**B**) PCL particles obtained after grinding and (**C**) 3D printed fibers and disks.

Recently, various research groups have been intensively working on producing drug loaded medical devices. Additionally, many methods have been used for drug incorporation into the carrier polymer. One of these methods is powder mixing of polymer and API [7]. As was mentioned before, the use of moderate particle sizes ranging from 250 to 600 µm should provide a homogeneous distribution of the drug in the polymer matrix [22]. The GS powder used in the current study had a regular spherical shape with an average size below 100 µm. These spheres were hollow as some ruptured ones were observed (Figure 3A). The particles of PCL obtained after grinding of pellets had an irregular shape, as shown in Figure 3B. The used grinder crushes the polymer pellets by forcing them into a sieve with a 750 µm diameter via centrifugal force. Thus, the produced particles should have a diameter smaller than 750 µm. This size was in the optimal range indicated by Saviano et al. [22].

A mixture of drug and polymer was used for preparing 3D printed samples with the theoretical drug concentration of 2.5% and 7.5% (*w/w*) (marked p2.5 and p7.5, respectively). Also, the samples printed from solvent cast materials with the same theoretical drug content (s2.5 and s7.5) were obtained. As a reference, samples made of pure PCL (pellets as received) were printed (Figure 3C).

The material was fed into the 3D printer and a preheating of 10 min at 100 °C was applied to assure the PCL melting. This time was also long enough to plasticize polymer-drug mixtures. PCL has a low melting point of 60 °C [33]. Thus, 100 °C was selected for the printing process, as it is higher than the melting point of the polymer and good flow characteristics through the nozzle were achieved.

The obtained fibers were measured by a digital caliper and had a diameter of 0.92 ± 0.02 mm. The beginning of the fiber was printed on part of the printing platform while the middle section was hung in the air to achieve a circular cross section as shown in Figure 2. Thus, the fibers were in slight tension which caused a slight decrease in their diameter compared to the inner diameter of the nozzle. Samples for compression testing, which were cut from 3D-printed structures with 100% fill, had a diameter and height of 3.87 ± 0.05 mm and 3.97 ± 0.15 mm, respectively. The drug loading and drug release samples had a diameter and height of 9.78 ± 0.08 mm and 1.94 ± 0.18 mm, respectively. Their weight was around 135 ± 10 mg.

### 3.1. Thermal Properties

**Figure 4:**
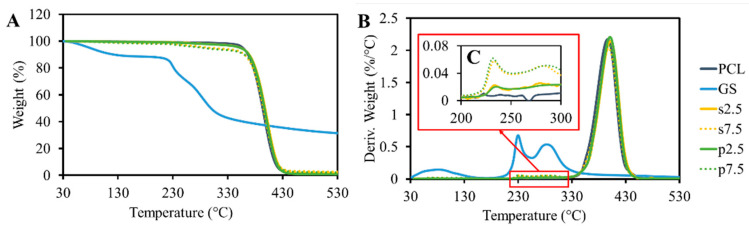
(**A**) TGA analysis of the produced PCL-GS samples, weight loss, (**B**) derivative weight loss, and (**C**) GS thermal degradation region.

The results of the TGA analysis are shown in Figure 4. GS itself shows content moisture as 15% of weight loss, which occurred up to 130 °C. This was also reported by Rosenkrantz et al. [9]. After the water evaporation, the drug did not show any significant weight loss until 230 °C. At this temperature, a sudden decrease in weight was observed. These results demonstrated that the temperature of thermal degradation of GS is much higher than the processing temperature. The TGA results of pristine PCL showed that the polymer is thermally stable up to 400 °C. A peak was observed at the drug degradation temperature (Figure 4C). This peak is proportional to the drug loading amount in the samples. Moreover, no differences were observed when comparing the drug mixing approaches. On the other hand, the pure PCL samples did not show any peak in this region.

**Figure 5:**
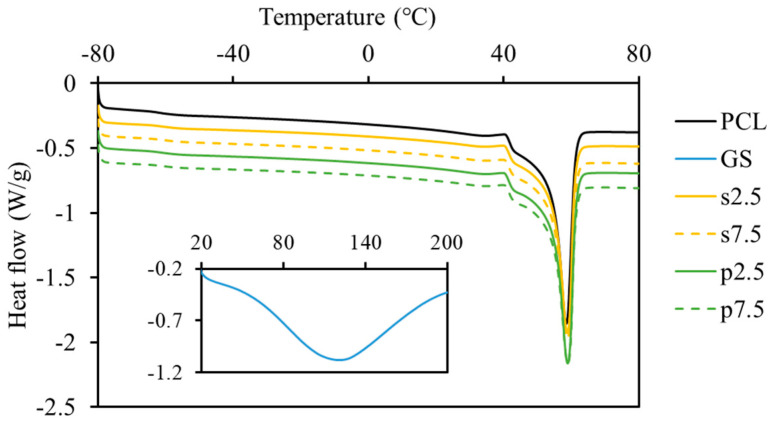
DSC analysis of produced PCL-GS samples (curves were shifted by −0.1 W/g). The insert presents the curve for GS.

DSC testing for the drug shows that there is no melting behavior between 0 °C and 200 °C for GS (Figure 5). However, a broad endothermic peak caused by the dehydration effect was present in the signal [9]. Moreover, DSC measurements showed that the loaded drug had no significant effect on the melting temperature (Figure 5 and Table 1). The crystallinity of the produced samples, which was calculated for the real polymer content, did not show any differences between the groups as no effect was caused by the presence of the drug particles (Table 1).

### 3.2. Mechanical Properties

**Figure 6:**
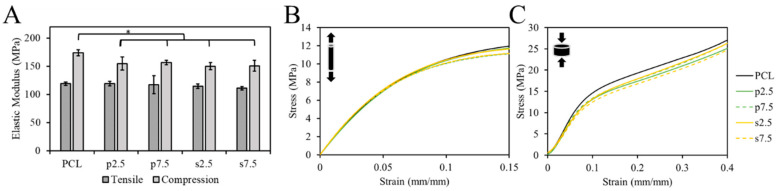
(**A**) Calculated elastic modulus of tensile and compression test (* *p* < 0.05) and representative Stress-Strain curves for (**B**) tensile and (**C**) compression test for PCL and PCL loaded with GS samples.

The mechanical properties of samples were tested using the tensile and compression test. The EM was used to compare groups. The single fibers were used for the tensile test. The pure PCL exhibited an EM of 119 ± 3 MPa. For the drug loaded samples, it was 119 ± 4, 117 ± 16, 114 ± 4 and 111 ± 3 MPa for p2.5, p7.5, s2.5 and s7.5, respectively. As shown in Figure 6A, there was no statistically significant difference in the tensile EM between the pure PCL and drug loaded samples, nor between the two mixing approaches in the fibers. This suggests that the addition of the drug up to 7.5% (*w/w*) had no effect on the tensile mechanical properties of the produced fibers. Thus, such low concentrations had no effect on the integrity of the material. This corroborates with the results of the DSC test; no significant change in the crystallinity nor T_m_ was observed suggesting a lack of changes in the microstructure of PCL matrix.

On the other hand, 3D printed samples were used in the compression test. The compressive EM showed a statistically significant difference between the pure PCL and the drug loaded PCL in a slight decrease of c.a. 13 ± 3% in the modulus in the 3D printed structures (Figure 6). However, no difference between the two mixing approaches was observed. The compressive EM of the pure PCL samples was 174 ± 5 MPa. For the drug loaded samples, it was 155 ± 12, 157 ± 4, 150 ± 6 and 151 ± 10 MPa for p2.5, p7.5, s2.5 and s7.5, respectively. Similar results for 3D printed samples were reported by Tappa et al. [34] when loading polylactic acid (PLA) with GS. They loaded PLA with GS at 2.5% (*w/w*) and observed a decrease in the compression EM by 48%.

### 3.3. Drug Distribution

**Figure 7:**
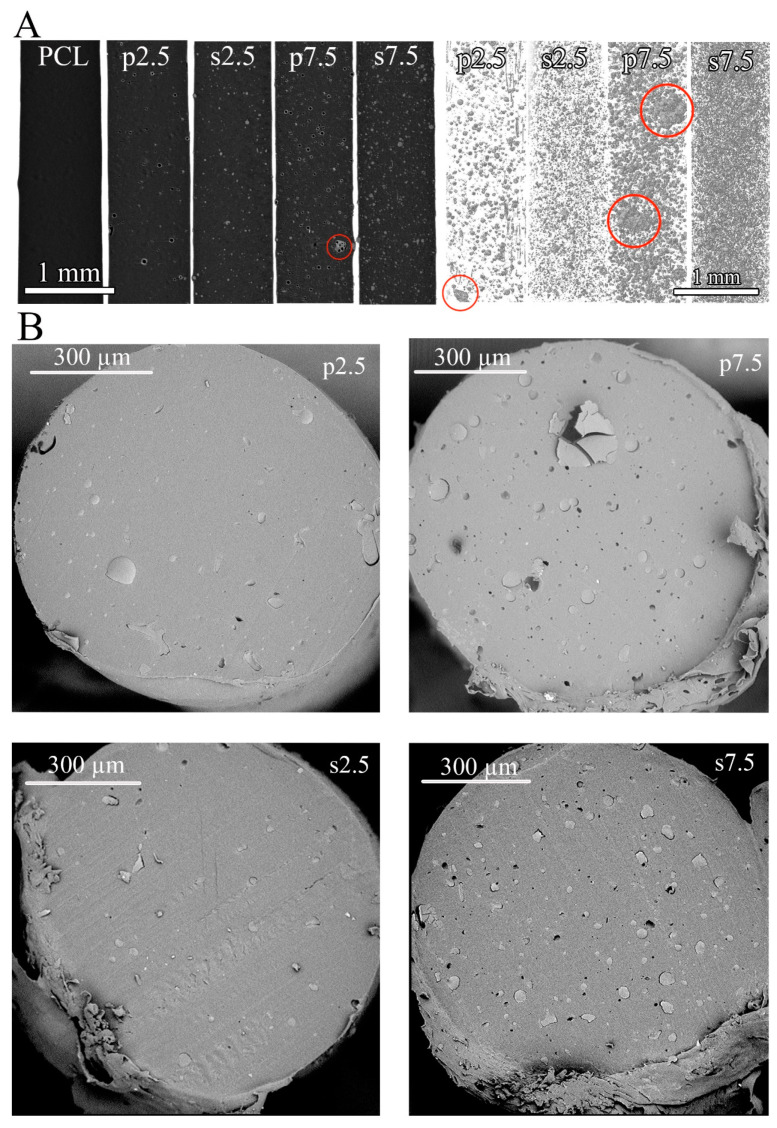
(**A**) Reconstructed cross sections of scanned fibers using µCT scanner (**left**) and 3D models showing only drug particles (**right**), red circles indicate agglomerates of GS within the PCL matrix and (**B**) SEM visualization of a cross sections prepared by cryosectioning from the printed fibers.

The drug distribution within the polymeric matrix after 3D printing was evaluated by two techniques: SEM and μCT. The dispersion of GS particles in the PCL varied when comparing the two mixing approaches. Figure 7 shows 3D reconstruction of the scanned fibers and SEM visualizations of the cross sections of fibers. The main difference between the two mixing approaches is the shape of the GS particles. It was observed that in the powder mixing approach, the GS shape was not altered during processing and the hollow spheres were visible in the μCT reconstruction (both 3D and cross section) (Figure 7A). On the other hand, the shape of the GS was altered and smaller solid particles were observed in the solvent casting approach (Figure 7A). This indicates that during the solvent casting approach the shell structure of GS was destroyed during the stirring process. Additionally, some agglomerates were visible in the powder mixing approach indicated by red circles in Figure 7A. Such agglomerates were not present in the solvent casting approach and the drug distribution was much more homogenous. 3D reconstructions of fibers printed from pristine PCL showed no pores in the produced fibers.

Quantitative data calculated from the μCT reconstructions are shown in Figure S1. As can be seen in Figure S1A, the GS particles’ dimension shows similar trend in both the powder mixing and the solvent casting approaches regardless of the drug loading. However, when comparing the two approaches, the quantitative data shows differences in particles’ dimensional distribution which confirms the visual observations shown in Figure 7A. Around 60% of the total volume of GS particles has a dimension below than 16 μm and 22 μm in the solvent casting and the powder mixing approaches, respectively (Figure S1B). Moreover, the average GS particles’ dimension is 18.42 ± 2.42, 17.09 ± 0.63, 28.83 ± 13.93 and 21.09 ± 5.70 μm for s2.5, s7.5, p2.5 and p7.5, respectively (Figure S1C). It can be concluded that the solvent casting approach produced smaller drug particles with smaller standard deviation. On the contrary, the powder mixing approach produced larger drug particles (around 1.5 times) with larger standard deviation.

SEM images of cross sections of the single fibers shown in Figure 7B confirmed the results obtained by the µCT reconstruction in terms of drug distribution and particle size. However, in the SEM images the immiscibility of GS in the PCL matrix was much more apparent. This was found in all drug loaded groups.

### 3.4. Drug Loading and Release Profile

**Figure 8:**
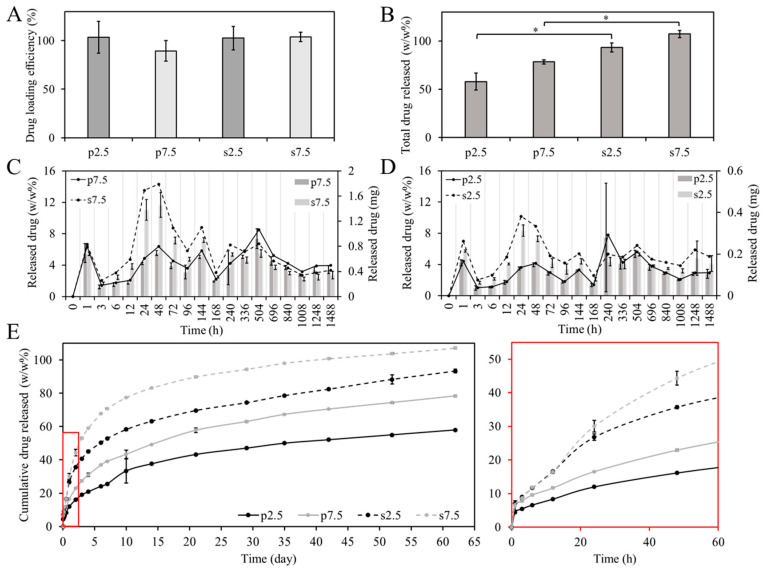
(**A**) Drug loading efficiency (ratio between the theoretical to the measured loading) in both mixing approaches; (**B**) Total amount of released drug expressed in the percentage of the (*w/w*) ratio after 62 days (* *p* < 0.05), released drug expressed in the percentage of the (*w/w*) ratio (lines, left axis) and measured mass (bars, right axis) for samples with (**C**) 7.5%, and (**D**) 2.5% of loaded drug and (**E**) Cumulative drug released (*w/w*) from prepared samples for 62 days (for drug release test n = 3).

The drug loading efficiency is a very important parameter, especially from an economical point of view. The most desired are the technologies that provide a level close to 100%. In our studies, around 100 ± 11% of the GS was detected from the dissolved samples. The drug loading efficacy test showed that there was no statistically significant difference between the two mixing approaches in terms of drug loading efficiency as shown in Figure 8A. However, there was an observed difference in the release profile. As shown in Figure 8B, most of the loaded drug (around 90–100% of the loaded drug) was released from samples prepared by the solvent casting approach, while only 50–60% of the loaded drug was released from samples prepared by powder mixing after 62 days of release. This could be due to the difference in distribution of drug particles as illustrated in Figure 7. Moreover, the higher release can be explained due to the smaller drug particles. This contributed to the faster dissolution of the drug particles.

Moreover, Figure 8C,D shows the weight percentage and the mass of the released drug at each time point. It can be noticed that most of the time points have a relatively similar release when comparing between the two mixing approaches, except for time points 24 and 48 h. During this period, it is expected that the PBS would have diffused into the whole volume of the sample. At these two times points, a relatively higher release was observed from samples prepared by the solvent casting approach at the two drug loading concentrations. At 2.5% drug loading, the release was around two times higher in the solvent casting at the two time points. At 7.5% drug loading, the release was around three and two times higher in the solvent casting at 24 h and 48 h, respectively. This caused the inflection in the drug release curve shown in Figure 8E. Furthermore, the shift in the drug release curve illustrated in Figure 8E between the two mixing approaches is almost the same between the two loading concentrations (2.5% and 7.5%). This demonstrates that the mixing approach affects the drug release profile. To better understand the release kinetics of each system, the release data were fitted into three commonly used models: (1) First order fitting, (2) Korsmeyer–Peppas model and (3) Higuchi model. The equations representing each model are presented in the supplementary material (Equations (S1)–(S3)). As can be seen from supplementary Figure S2 and Table S1, the Korsmeyer–Peppas model provides the best R^2^ values. Thus, this model was selected to interpret the release kinetics. In this model the values of n represent the diffusion component which describe the drug release kinetics. For disk shaped samples, the n value for a Fickian diffusion mechanism should be located around 0.5 [35,36]. As can be seen in the supplementary Table S1, the n values obtained from the fitting were 0.42, 0.52, 0.39 and 0.38 for s2.5, s7.5, p2.5 and p7.5, respectively. These values indicate a Fickian diffusion release kinematic.

### 3.5. Antibacterial and Preventive Activity on Biofilm Formation

As pointed out in the introduction, the potential application for these 3D printed materials would be the generation of delivery systems to prevent biofilm-related infections. This delivery systems are of special interest for prevention of bone infection as these avascular areas are unreachable for systemic antibiotics [37]. Because of this, the antibacterial and anti-biofilm properties of both the materials and eluates of the different time points was assessed. All eluates from all time points showed antibacterial activity against *S. aureus* (ATCC 25923). When comparing the two mixing methods, MIC values for the eluates confirmed the higher release from the samples corresponding to the solvent casting at the time points 24 and 48 h (Figure 8C,D). This was demonstrated by testing the antibacterial properties of the extracts after several dilutions, as shown in Table 2. At 2.5% (*w/w*), drug loading eluates showed two-fold higher antibacterial properties for samples prepared by solvent casting at both time points. On the other hand, at 7.5% (*w/w*) the drug loading eluates showed 4- and 2-fold higher antibacterial properties at time points 24 h and 48 h, respectively. This corroborates with the results obtained in the drug release measurements.

The effect of the eluates on the prevention of the biofilm formation as well as the anti-biofilm capability of the materials was also studied. It is essential to confirm the antimicrobial capability of both the eluates and the materials, to guarantee not only a prevention of biofilm formation onto the material but also in the surrounding tissue.

**Figure 9:**
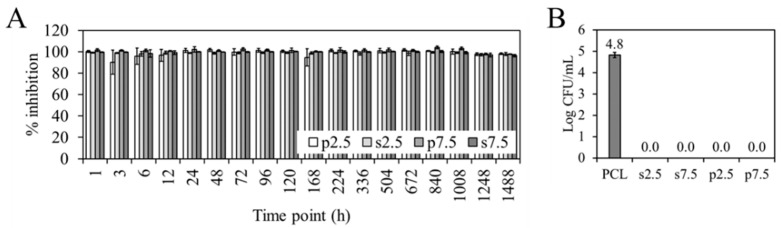
(**A**) Effect of the eluates on biofilm formation, percentage of inhibition calculated with respect to the bacterial control, (**B**) Number of viable attached *S. aureus* (ATCC 25923) (log CFU/mL) on PCL printed samples after 24-h incubation. Results expressed as mean ± SD of two biological replicates, with three technical replicates.

No differences were found in terms of prevention of biofilm formation between the two mixing approaches. Figure 9A shows the percentage of inhibition of the biofilm formation of the different time point eluates, calculated with respect to the biofilm formed by *S. aureus* (ATCC 25923) in the absence of eluate (bacterial control). All the eluates inhibited the biofilm formation for more than 90%. This means that the concentration of GS in all the eluates is above the Minimum Biofilm Inhibitory Concentration (MBIC). This refers to the concentration needed for a compound to cause a 90% inhibitory effect on bacteria when a biofilm is developing [38].

Figure 9B show the viable attached *S. aureus* (ATCC 25923) to the different materials. It can be seen how both approaches and both dosages completely prevent bacterial adherence when compared to the PCL control.

Our results are promising towards a success in the clinic, as both approaches provide a concentration of GS above the MBIC up to two months. Stravinskas, M et al. [39] studied if the in vitro release test of a synthetic bone graft loaded with GS predicted the performance of the material in vivo. In their study, the release of GS above the MIC for 28 days correlated with prevention of bone infection in the clinic. In our study, we have shown how both approaches guarantee a concentration of gentamicin not only above the MIC but also above the MBIC. However, it has to be taken into account that therapeutically, much higher concentrations are needed to reach bactericidal effect (four to eight times the MIC [40]). In that sense, the solvent casting would be a better approach to guarantee an efficient antimicrobial and anti-biofilm activity, as it has shown a significant higher release of the drug.

## 4. Conclusions

The production of drug loaded devices using 3D printing has shown promising results over the past few years. However, as most available 3D printers do not offer good mixing steps prior to printing, it is very important to prepare a homogenous mixture of the polymer and drug before loading it into the 3D printer. In this study, two commonly used approaches were compared, namely powder mixing and solvent casting. The material preparation steps varied among the two approaches. Powder mixing can be considered a more straight-forward process in case the polymer was supplied in a powder form. This is advantageous when moving towards industrialization. On the other hand, the solvent casting approach requires more steps. Additionally, the need for organic solvent can be considered a disadvantage in this process as using such solvent in industrial scale is not considered environmentally friendly. However, the solvent casting approach provided a more homogenous mixture with higher drug dispersing abilities compared to powder mixing. In this process the spherical shape of the drug particles was altered. Both mixing approaches resulted in similar thermal and mechanical properties. However, in the compression test the drug loaded samples in both approaches showed a decrease in the mechanical properties. The effect of drug loading on the mechanical properties of 3D printed constructs needs more investigation which can be considered in future work. Moreover, the drug release profile showed a significant difference. Samples prepared by solvent casting exhibited higher release characteristics. Despite both approaches produced efficient antibacterial and anti-biofilm materials, the solvent casting approach displayed a higher antimicrobial effect due to the higher release.

## Figures and Tables

**Table 1:** DSC analyses results of produced PCL-GS samples.

	PCL	p2.5	p7.5	s2.5	s7.5
**T_m_ (°C)**	58.8 ± 0.3	59.3 ± 0.4	59.1 ± 0.1	58.9 ± 0.2	59.1 ± 0.2
**Crystallinity (%)**	41.5 ± 0.2	42.4 ± 0.2	41.4 ± 1.2	41.6 ± 0.6	42.9 ± 1.1

**Table 2:** Number of dilutions performed on eluates produced from the drug release test at time points 24h and 48h that still inhibit *S. aureus* (ATCC 25923) growth. Results corresponding to three biological replicates, with three technical replicates.

	s2.5	p2.5	s7.5	p7.5
**24 h**	1:4	1:2	1:16	1:4
**48 h**	1:4	1:2	1:16	1:8

## Supplementary Materials

The following are available online at https://www.mdpi.com/1996-1944/13/15/3364/s1, Figure S1. (a) Range frequency calculated as a percentage volume in a certain dimensional range, with (b) illustrating the cumulative frequency highlighting when percentage volume reaches around 60% of the total volume and (c) Particles’ average dimension at different drug loading percentages, Equations S1–S3: illustrating models to describe the release kinetics, Table S1: Results from curve fitting the release curves to different models illustrating the value of each model parameters and the R^2^ values. The model with the highest R^2^ is highlighted, Figure S2. Release kinetics of GS from PCL samples according to (a) First order fitting, (b) fitting to Korsmeyer-Peppas model and (c) fitting to Higuchi model.
