# Supplementary material for: 3D-Printed Drug Delivery Systems: The Effects of Drug Incorporation Methods on Their Release and Antibacterial Efficiency
Source: Materials (Basel). 2020 Jul 29;13(15):3364. doi: 10.3390/ma13153364 (PMC7435804; doi:10.3390/ma13153364)
Supplement: Supplementary file 1 [file materials-13-03364-s001.pdf]

3D-Printed Drug Delivery Systems: The Effects of Drug Incorporation Methods on Their Release and Antibacterial Efficiency

Bahaa Shaqour, Inés Reigada, Żaneta Górecka, Emilia Choińska, Bart Verleije, Koen Beyers, Wojciech Święszkowski, Adyary Fallarero and Paul Cos

Microcomputed topography quantitative measurements

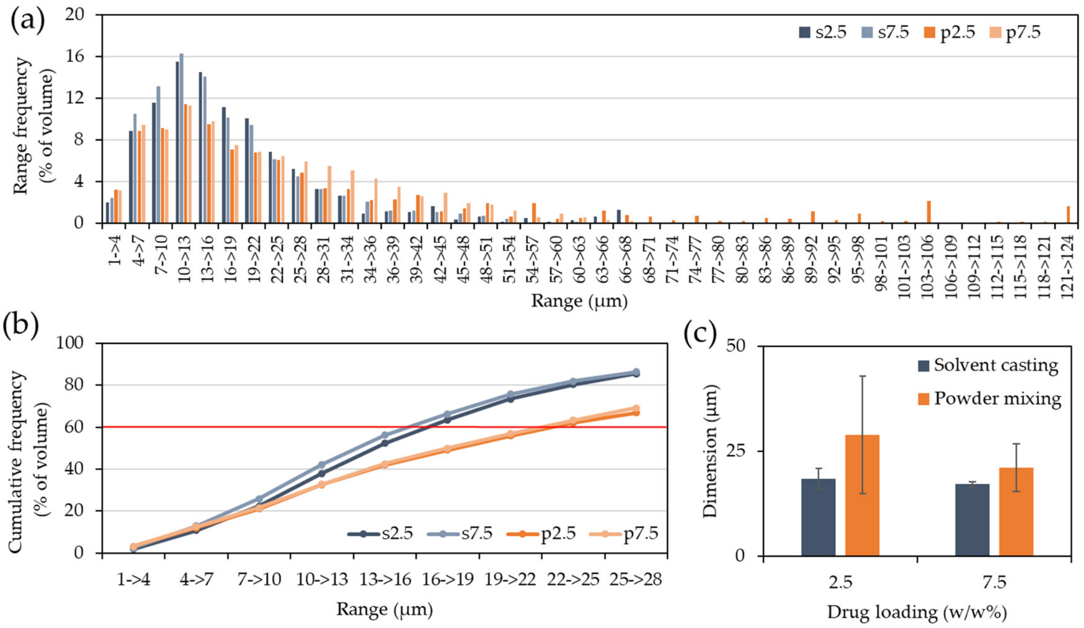

Figure S1. (**a**) Range frequency calculated as a percentage volume in a certain dimensional range, with (**b**) illustrating the cumulative frequency highlighting when percentage volume reaches around 60% of the total volume and (**c**) Particles' average dimension at different drug loading percentages.

Release kinetics models

1. First order kinetics:

$$\log\left(100 - \frac{m_t}{m_\infty}\%\right) = -\frac{K}{2.303}t \qquad \text{(S1)}$$

where m_t is the released amount at a certain time point, m_∞ is the amount of the drug released after an infinitive time, K is the first-order release rate constant and t is time [1].

2. Korsmeyer-Peppas model:

$$\log\left(\frac{m_t}{m_\infty}\%\right) = \log k_{K-P} + n \log t \qquad \text{(S2)}$$

where m_t is the released amount at a certain time point, m_∞ is the amount of the drug released after an infinitive time, k_{K-P} is the Korsmeyer-Peppas constant, n is the parameter indicative of the drug release mechanism and t is time [1]. The release model is fitted up to 60% of the release data.

3. Higuchi model:

$$Q = k_H t^{0.5} \quad \text{(S3)}$$

where Q is the percentage of the released drug, k_H is the Higuchi rate constant and t is time [1].

Table S1. Results from curve fitting the release curves to different models illustrating the value of each model parameters and the R^2 values. The model with the highest R^2 is highlighted.

Model	Formulation	Parameters		R^2
First order	s2.5	$K = 0.0016$		0.9603
	s7.5	$K = 0.0041$		0.9816
	p2.5	$K = 0.0005$		0.9084
	p7.5	$K = 0.0009$		0.9484
Korsmeyer-Peppas	s2.5	n = 0.4235	$k_{K-P} = 6.2302$	**0.9857**
	s7.5	n = 0.5219	$k_{K-P} = 5.3815$	**0.9758**
	p2.5	n = 0.3856	$k_{K-P} = 3.6568$	**0.9921**
	p7.5	n = 0.3820	$k_{K-P} = 5.2808$	**0.9876**
Higuchi model	s2.5	$k_H = 2.2190$		0.9357
	s7.5	$k_H = 2.6932$		0.8744
	p2.5	$k_H = 1.4972$		0.9766
	p7.5	$k_H = 1.9800$		0.9753

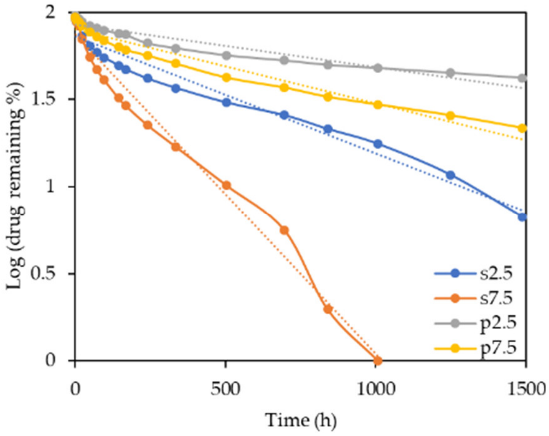

(a)

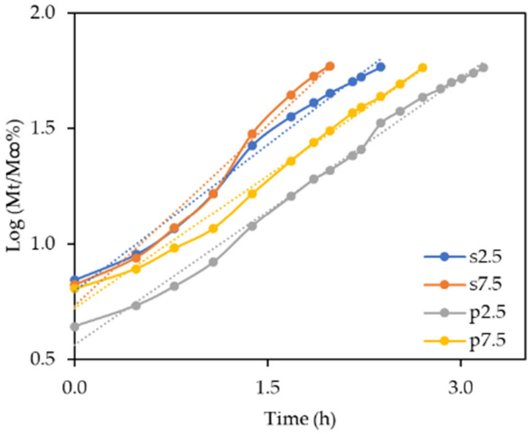

(b)

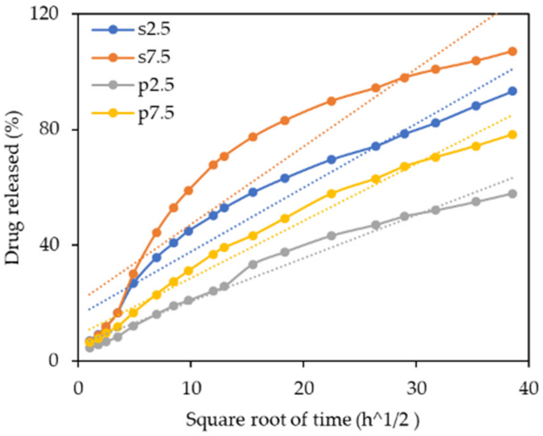

(c)

Figure S2. Release kinetics of GS from PCL samples according to (**a**) first order fitting, (**b**) fitting to Korsmeyer-Peppas model and (**c**) fitting to Higuchi model. Note: In first order modeling of s7.5 samples data points were limited to 1008 hours as the drug release reached 100%.

References

1. S., K.; Rama Pawar, R.; D. Kevadiya, B.; C. Bajaj, H. Synthesis of Saponite Based Nanocomposites to Improve the Controlled Oral Drug Release of Model Drug Quinine Hydrochloride Dihydrate. *Pharmaceuticals* **2019**, *12*, 105, doi:10.3390/ph12030105.
